# The Comorbidities Coma Scale (CoCoS): Psychometric Properties and Clinical Usefulness in Patients With Disorders of Consciousness

**DOI:** 10.3389/fneur.2019.01042

**Published:** 2019-10-17

**Authors:** Francesca Pistoia, Antonio Carolei, Yelena G. Bodien, Sheldon Greenfield, Sherrie Kaplan, Simona Sacco, Caterina Pistarini, Alfonsina Casalena, Antonio De Tanti, Benedetta Cazzulani, Gianluca Bellaviti, Marco Sarà, Joseph Giacino

**Affiliations:** ^1^Department of Biotechnological and Applied Clinical Sciences, Neurological Institute, University of L'Aquila, L'Aquila, Italy; ^2^Department of Physical Medicine and Rehabilitation, Spaulding Rehabilitation Hospital-Harvard Medical School, Boston, MA, United States; ^3^Department of Neurology, Massachusetts General Hospital, Boston, MA, United States; ^4^Health Policy Research Institute, University of California, Irvine, Irvine, CA, United States; ^5^Salvatore Maugeri Foundation, Scientific Institute of Nervi, Nervi, Italy; ^6^Hospital G. Mazzini, Teramo, Italy; ^7^Cardinal Ferrari Rehabilitation Centre, Fontanellato, Italy; ^8^Salvatore Maugeri Foundation, Scientific Institute of Pavia, Pavia, Italy; ^9^Post-Coma Rehabilitative Unit, San Raffaele Hospital, Cassino, Italy

**Keywords:** coma, vegetative state, unresponsive wakefulness syndrome, minimally conscious state, comorbidities

## Abstract

Although comorbidities have a well-known impact on the functional recovery of patients with disorders of consciousness, including coma, vegetative state (VS), and minimally conscious state (MCS), a specific tool for their assessment in this challenging group of patients is lacking. For this aim, a multistep process was used to develop and validate the Comorbidities Coma Scale (CoCoS) in a sample of 162 patients with a diagnosis of coma, VS or MCS admitted to four Acute Inpatient Rehabilitation Units. To establish the psychometric properties of the scale, content validity, and internal consistency were investigated through Exploratory Factor Analysis in the whole sample (*n* = 162). Interrater reliability, assessed by the weighted Cohen's kappa (Kw), and concurrent validity of the scale as compared to the Greenfield Scale, assessed by ρ Spearman's correlation coefficient, were investigated in a subsample of patients (*n* = 52) within two of the above units. Our findings provided evidence of a good content validity of the scale, with the identification of a 12-factor structure representing the different comorbid dimensions of the target population. Inter-rater reliability was excellent in both the rehabilitation units where the assessment was made [Kw 0.98 (95% CI 0.96–0.99)]. CoCoS total scores correlated significantly with total scores of the Greenfield Scale (ρ = 0.932, 95% CI 0.89–0.96; *P* < 0.0001) indicating that CoCoS has concurrent validity while being more informative about the specific pattern of comorbidities of these challenging patients. The CoCos is a new tool which standardizes the approach to assessment of comorbid conditions and reliably identifies the category and severity of each comorbidity detected. It may be used for both clinical and research applications.

## Introduction

Disorders of consciousness (DOCs) include coma, vegetative state (VS) [also known as the Unresponsive Wakefulness Syndrome (UWS)] and minimally conscious state (MCS) (1–4). Coma is characterized by the lack of both wakefulness and awareness while patients in VS retain wakefulness but lack awareness (1–3). In MCS, there is inconsistent but clearly-discernible evidence of conscious awareness (4). Coma, VS and MCS occur along a clinical continuum: patients recovering from a coma may regain full consciousness quickly or may enter a VS, which, may represent the first step in the transition from coma to regaining consciousness or may persist indefinitely. Similarly, MCS may be a stage on the road to recovery of full consciousness or may become a chronic condition. Conversely, patients may decline when there are clinical complications or severe medical conditions persistently leading to a worsening of behavioral responsiveness.

Establishing a prognosis in patients with DOCs is very challenging in view of limited knowledge of the neural correlates of consciousness, extreme variability in patterns of brain injury, and the presence of multiple medical comorbidities associated with the primary brain injury. Recent studies have reported that both acute complications and chronic medical comorbidities are highly prevalent in patients with DOCs and strongly influence survival, recovery of consciousness, and functional independence (5–7). The most frequent complications and comorbidities involve the respiratory, urinary tract, and cardiovascular systems, with respiratory diseases and arrhythmias being reported as negative predictors of full recovery of consciousness and functional improvement (6, 7).

A validated assessment tool designed specifically to identify and quantify the burden of comorbidities on recovery in patients with DOCs is lacking. The use of a specific scale, targeted to the clinical characteristics and needs of patients with DOCs, would improve surveillance of comorbidities and inform the impact of associated conditions on diagnosis, prognosis, and outcomes. An important limitation of existing comorbidity assessment scales is that they have been designed for patients with other diagnoses, such as cancer and age-related diseases, whose spectrum of comorbidities differs from those most frequently encountered in patients with acute brain injury. Consequently, comorbidities that occur with higher frequency in DOCs vs. other complex disorders may be missed while those that are common to other diseases may be absent or rare in patients with DOCs and their assessment may be redundant and time consuming. Examples of comorbidities which occur more frequently in DOCs vs. age-related diseases and other complex diseases include dysautonomia, seizures, hydrocephalus, heterotopic ossification while medical conditions which are less common in patients with DOCS include depression, consequences of recurrent falls, hearing or visual loss, chronic arthritis, degenerative cognitive impairment. Some, like depression and previous cognitive impairment, are at risk of being underestimated due to patients' unresponsiveness. The use of a standardized and validated scale, targeted to the clinical characteristics and needs of patients with DOCs, would improve surveillance of comorbidities, inform the impact of associated conditions on diagnosis, prognosis, and outcomes, and support future research efforts. The implementation of a standardized assessment tool for comorbidities in DOCs would also improve the efficiency and consistency of the approach to assessment, increase the objectivity and reliability of the findings and enhance communication among clinicians of different countries, paving the way for prognostic and comparative studies.

The objective of the present study is to develop a new scale, the Comorbidities Coma Scale (CoCos), to detect comorbidities in patients with DOCs and to determine its psychometric properties.

## Materials and Methods

### Description of the Final Version of the CoCos

The CoCos includes 24 categories addressing the frequency of various comorbidities common in DOCs. For each category, scoring is based on the presence/absence of specific comorbidities and their severity. Disease categories include respiratory and urinary tract infections, non-infectious respiratory diseases, organic heart diseases, rhythm disorders without organic heart diseases, arterial hypertension, diabetes mellitus, dysautonomia, peripheral artery or venous diseases, supra-aortic trunks disease, hepatobiliary and gastrointestinal disorders, seizures, hydrocephalus, fractures and joint diseases, anemia, presence of life support devices, pressure ulcers, malignancies, malnutrition, renal diseases, and previous disability. Categories that have been included in the scale are described in Table 1. The severity of each disease category is hierarchically classified. As examples, the severity of respiratory infections is graded on the basis of the absence/presence of symptoms, the need for treatment and the response to the treatment: poor response denotes the most severely scored forms of the disease like treatment resistant infections and conditions evolving into septic shock. The severity of anemia is scored on the basis of the hemoglobin level and the need for transfusion while the severity for the need of life-support devices is scored by considering the number of devices (tracheotomy tube, nasogastric tube, percutaneous endoscopic gastrostomy, urinary catheter, central venous catheter), which are needed. The administration of the scale requires review of the medical history, clinical assessment, physical examination, and instrumental and laboratory tests, where necessary. The latter include blood and urine tests, radiographic and ultrasound examinations, brain computed tomography, magnetic resonance imaging, and electroencephalography. The CoCoS was developed to be used prospectively, by combining actual physical examination with screening of medical records or to accommodate retrospective assessment of comorbidities through comprehensive evaluation of medical records. The data-sheet for the CoCos also includes a box for each comorbidity that enables the examiner to indicate whether each comorbidity, when present, was pre-existing or newly diagnosed after the brain injury that caused the consciousness impairment, requiring management in the Acute Inpatient Rehabilitation Units. The former include comorbidities that occurred in the years preceding the onset of the index event while the latter include comorbidities that occur either concurrently with the injury causing the consciousness impairment (index event) or in the time period from the date of the index event to the date of comorbidities data collection. In the case the same comorbidity (for instance respiratory infection) occurred both in the years preceding the index event (thus belonging to the past medical history of the patient) and following the index event (thus belonging to the recent medical history of the patient), the examiner can point-out this scenario by choosing the specific option in the aforementioned box. In the case the clinical history of the patient it not enough detailed to report information about an item, the examiner should record the score as untestable choosing the specific option and clearly write the explanation for this choice in the box for comments. The cumulative comorbidity burden for each patient is denoted by the final summative score ranging from 0 (no comorbidities) to 72 (presence of all comorbidities at maximum of their severity). Patients are stratified into three severity categories based on the final cumulative score using the following proposed cut-off scores: (0: no comorbidities; range 1–24: presence of mild comorbidities; range 25–48: moderate comorbidities; range 49–72: severe comorbidities). In the case of missing untestable data, to avoid inaccurate computation of the total cumulative score and inappropriate attribution to one of the three severity categories, total score will not be taken into account whether more than two variables turn out to be untestable.

**Table 1:** Description of disease categories included in the CoCoS.

Respiratory infections	Upper Respiratory Infections like sinusitis, pharyngitis, epiglottitis, and raryngotracheitis Lower Respiratory Infections like bronchitis, bronchiolitis, and pneumonia
Urinary tract infections	Cystitis, Pyelonephritis, Asymptomatic Bacteriuria, Renal Abscess
Non-infectious respiratory diseases	Chronic obstructive pulmonary disease, pulmonary edema, respiratory failure
Organic heart diseases	Stable and unstable angina, myocardial infarction, severe coronary atherothrombosis, valvulopathies, endocarditis, myocarditis, pericarditis, congestive heart failure, arrhythmias associated with one or more of the above diseases
Rhythm disorders without organic heart diseases	All cardiac arrhythmias that arise in the absence of structural heart diseases
Arterial hypertension	Primary or secondary hypertension
Diabetes mellitus	Evidence of glucose intolerance or diabetes
Dysautonomia	Paroxysmal tachycardia, tachypnea, increased systolic blood pressure, hyperthermia or hypothermia, excessive sweating, signs of decerebration or decortication, increased muscle tone, horripilation, flushing
Peripheral artery disease	Evidences of peripheral artery disease
Supra-aortic trunks disease	Unilateral or bilateral carotid stenosis
Peripheral venous disease	Evidence of peripheral artery occlusive disease
Hepatobiliary diseases	Cholelithiasis, viral or toxic hepatitis, cirrhosis, liver failure
Gastrointestinal disorders	Gastroesophageal reflux disease, peptic ulcer disease, diverticulitis
Seizures	Presence of sporadic seizures, recurrent seizures or status epilepticus
Hydrocephalus	Normal pressure hydrocephalus or hydrocephalus requiring a ventriculoperitoneal shunt and/or decompressive craniectomy
Fractures	Traumatic or pathological fractures
Presence of life-support devices	Presence of tracheotomy tube, nasogastric tube, percutaneous endoscopic gastrostomy, urinary catheter, central venous catheter
Anemia	Symptoms and signs of anemia
Joint diseases	Inflammatory or degenerative joint diseases
Pressure ulcers	Damage to the skin and/or underlying tissue resulting from prolonged pressure on the skin
Malignancies	History of any cancer (index disease excluded)
Malnutrition	Insufficient, excessive or imbalanced consumption of nutrients as inferred by physical parameters and laboratory findings
Previous disability	History of disability caused by a previous disease occurred before the injury responsible for consciousness impairment
Renal diseases	Nephrolithiasis, renal failure

*One category (cerebrovascular diseases meaning any recurrent event following the index disease) of the original 25-item scale was removed from the final version as a result of the low incidence*.

The full scale is provided in the Supplementary Material and the whole process of development of the scale is described in the following sections. The research protocol was approved by the Internal Review Board of the University of L'Aquila (no. 16/2019).

### Multistage Development of the CoCoS

The CoCos development process was carried out in different stages as suggested by existing practical recommendations on tool development.

**Figure 1:**
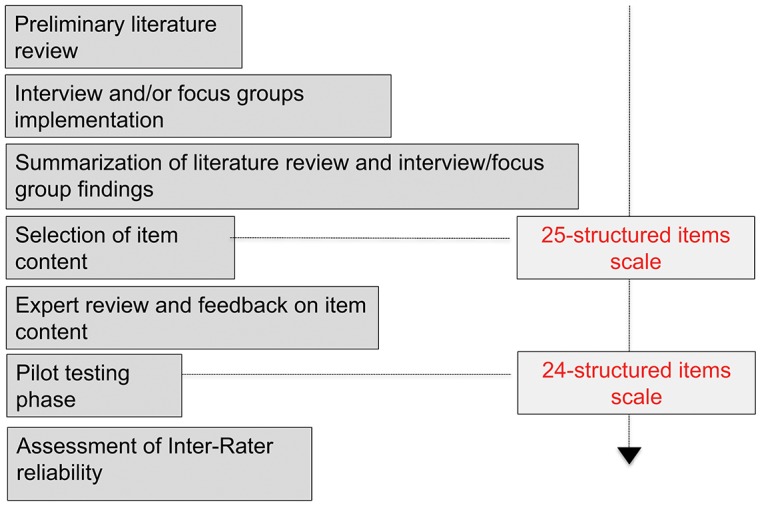
Multistage development of the CoCoS.

The design process included the following steps and was summarized in Figure 1:

1. Preliminary literature review
2. Interview and/or focus groups implementation with experts in medical management of DoC
3. Summarization of literature review and interview/focus group findings
4. Selection of item content, resulting in the 25-structured items scale
5. Expert review and feedback on item content
6. Pilot testing phase, resulting in the 24-structured items scale
7. Assessment of Inter-Rater reliability

#### Preliminary Literature Review

A preliminary literature review was performed in order to identify existing comorbidity scales or items, which could be used or adapted in the proposed new tool. The following key search terms were used: comorbidities, illness, scale, coma, vegetative state, unresponsive wakefulness syndrome, minimally conscious state, and consciousness. This ensured that the proposed tool was aligned with prior research on comorbidities and provided a framework for adding new categories to the CoCos to more reliably identify comorbid conditions and severities in patients with DOCs. The following published scales assessing comorbidities were reviewed: Greenfield's Scale for comorbidities (also known as the Index of Coexistent Disease), the Charlson Comorbidity Index, the Geriatric Index of Comorbidity, Adult Comorbidity Evaluation-27, Kaplan-Feinstein Index, Chronic Disease Score, Cumulative Illness Rating Scale, Multipurpose Australian Comorbidity Scoring System, Simplified Comorbidity Index, Total Illness Burden Index, Washington University Head and Neck Comorbidity Index, and Pharmacy-based Comorbidity Index (8–21). None of these instruments was specifically developed for patients with DOCs. However, they were carefully evaluated to ascertain whether they were able to capture the entire spectrum of comorbidities even in patients with DOCs and to identify to what extent the items could contribute to develop a new measure. Following careful evaluation of the measures above, it was concluded that the specific characteristics of patients with DOCs are not captured by the previously published scales. In fact, all the available instruments had been developed for different patient groups from those targeted in this study. As such, they include medical conditions that cannot be reliably assessed in patients with DOCs due to the barrier of unresponsiveness (such as depression and age-related hearing loss), and omit others that are commonly observed in this population (seizures, hydrocephalus, heterotopic ossification). Therefore, the development of an additional tool to assess comorbidities in patients with DOC was justified by the need to describe comorbidities that occur in the other populations as well as those that are unique to patients with DoC. Thus, the existing scales were reviewed to identify elements that could be incorporated into the CoCos.

#### Interviews and/or Focus Groups Implementation

Interviews and focus groups with experts in the medical management of DoC and health care professionals who were likely to become the main users of the proposed scale were carried out to obtain input regarding the content of the scale. This contributed to the process of conceptualization of the new construct with great attention being paid to the level of understanding of the proposed construct across different potential users among health care professionals.

#### Summarization of Literature Review and Interviews/Focus Groups Findings

At this point, findings from the literature review and interviews/focus were merged. When discrepancies between the two sources were present, including those about the potential redundancy of some items, the most comprehensive approach was adopted in order to not exclude any potentially useful dimensions of the construct.

#### Developments of the Scale's Items

Based on the literature review and focus groups clinicians and researches involved in the current study and with a specific and long-standing expertise in the management of patients with DOCs selected candidate items for inclusion in the CoCos. Items to be added were also identified based on data from previous studies investigating the presence and the prognostic role of comorbidities in patients with DOCs (5–7). The number and content of items included was informed by the overarching aim of designing a tool that was informative, valid, reliable, simple to administer, and time-efficient. Therefore, items that were judged to be non-contributory to the underlying construct were eliminated. When uncertainty remained about including specific items, most were retained with the possibility of deletion at later stages of the design process. Special attention was paid to drafting the items in a clear manner, and by specifying, whenever necessary, specific reference values for laboratory and imaging results. Response options for each item were selected using a modified Likert-type four-point response scale where 0 indicated the absence of any comorbidity and 3 represented the most severe form of the observed comorbidity. At the end of this stage, a consensus meeting was held to agree on a final version, with the aim of developing a scale that would be relevant and comprehensive while avoiding duplication or items. Problems with interpretation and relevance of items were discussed and resolved. This phase lead to the original 25-structured items scale.

#### Expert Review and Feedback

The scale was introduced and discussed at several national and international conferences to obtain feedback from experts in this field (22, 23). Specifically, representativeness (i.e., how completely the items address the construct of comorbidities in DoC), precision (i.e., how clearly the items are worded), relevance (to what extent each item actually contributes to specific aspects of comorbidities in DoC), and distribution of the items were discussed. Further feedback from experts was used to improve the content validity of the scale and to refine the items. At the end of this stage, consensus was reached on a scale (mainly derived from the Greenfield's Scale for Comorbidities) including 25 categories of comorbidities, each one hierarchically classified using a severity score ranging from 0 to 3.

#### Pilot Testing Phase

Pilot testing was performed to obtain preliminary data from the target population, to evaluate range and variance of the selected items and to identify items not contributing in a meaningful way to the final tool. Medical records of a large sample of patients admitted with a diagnosis of DOC in four Acute Inpatient Rehabilitation Units (Salvatore Maugeri Foundation, IRCCS, Pavia, Italy; Cardinal Ferrari Rehabilitation Centre, S. Stefano Institute, Fontanellato, Parma, Italy; San Raffaele Hospital, Cassino, Italy; Rehabilitation Center Sant'Agnese, Pineto, Italy) were retrospectively reviewed to obtain the CoCoS scores. The consciousness profile of patients had been evaluated through the CRS-R which is the only tool recommended for the assessment of DOC by the American Congress of Rehabilitation Medicine with minor reservations (24–26). The pattern of responses, reported as absolute numbers with percentages, mean ± standard deviation (SD), or median with interquartile range (IQR), were analyzed to determine the best distribution for modeling frequency counts. Later, advanced statistical techniques including exploratory factor analysis were performed to estimate the internal structure of the scale and the concurrent validity. All the analyses were performed by means the SPSS 20.0 software. This process contributed to the item calibration and lead, through the exclusion of one non-meaningful item (cerebrovascular diseases), to obtain the final 24-item scale.

#### Assessment of Reliability and Concurrent Validity of the CoCos

Inter-rater reliability of the final 24-item scale was investigated in a subsample of the original population. The CoCoS scores were obtained through a retrospective examination of medical records by two raters in two of the aforementioned centers. Medical records in both centers were also evaluated by a third examiner in order to obtain the Greenfield Scale scores (8) to evaluate concurrent validity. These examiners were blinded to the data previously collected by means of the CoCos. Interrater reliability was assessed, through the SPSS 20.0 software, by the weighted Cohen's kappa (Kw) while concurrent validity was evaluated by ρ Spearman's correlation coefficient using the Greenfield Scale as the available gold standard (albeit non-specifically developed for patients with DOC) and the CoCos as the test measure.

## Results

### Pilot Testing

#### Participants

The sample was comprised of 162 patients (97 men and 65 females, mean age 57.3 ± 17.6) with a history of acute traumatic or non-traumatic coma (defined by a Glasgow Coma Scale score of 3–8), and a diagnosis of coma, VS or MCS based on the Coma Recovery Scale—Revised (CRS-R) profile at the time of the enrolment (24). Patients with chronic DOC at admission (1 year duration from onset) or presenting acute complications requiring readmission to the Intensive Care Unit before enrolment were excluded.

The most frequent condition leading to loss of consciousness was stroke (*n* = 80; 49.4%), followed by traumatic brain injury (*n* = 47; 29.0%), anoxic damage (*n* = 29; 17.9%), and other diseases (*n* = 6; 3.7%). The most frequent clinical diagnosis was VS (*n* = 84; 51.9%) followed by MCS (*n* = 71; 43.8%) and coma (*n* = 7; 4.3%). The time-interval between the acquired brain injury and the admission to the Acute Inpatient Rehabilitation Units was between 30 and 60 days in 38% of patients, between 15 and 30 days in 32% of patients, more than 60 days in 27% of patients and <15 days in 3% of patients. The time to complete the CoCoS assessment for each patient was 10 ± 3.0 SD minutes.

#### Patterns of Distribution of Comorbidities

**Figure 2:**
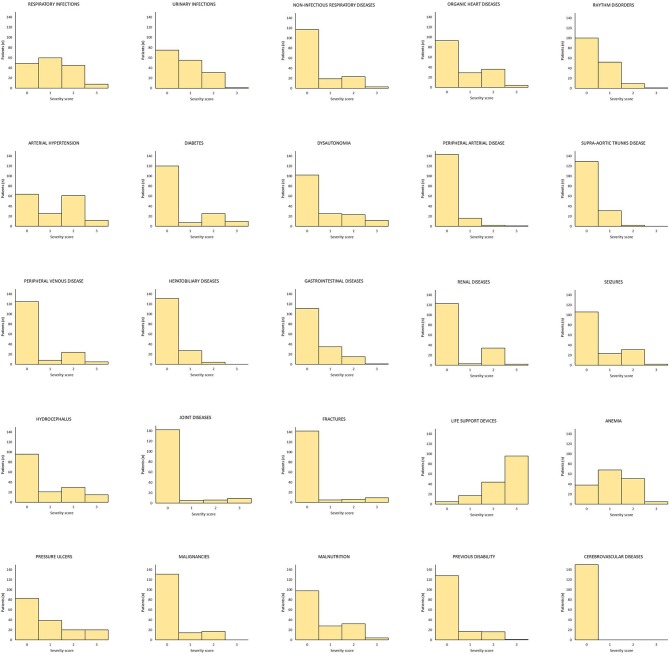
Distribution of frequency and severity scores of comorbidities in the original 25-item scale. The category “Cerebrovascular diseases” was excluded as a result of its low incidence.

**Figure 3:**
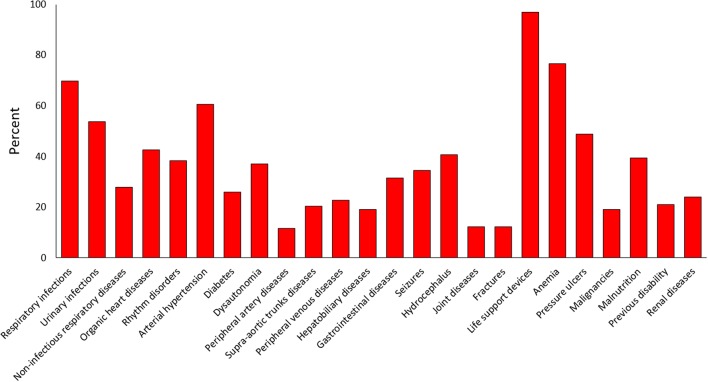
Frequency of comorbidities finally included in the scale.

The frequency and severity distribution of comorbidities is shown in Figure 2. All but five patients required at least one life support device (i.e., tracheostomy tube, nasogastric tube, percutaneous endoscopic gastrostomy, urinary catheter, central venous catheter etc). Anemia (*n* = 124; 76%, 95% CI: 69–82), respiratory infections (*n* = 113, 70%, 95% CI: 62–76), urinary tract infections (*n* = 75; 46%, 95% CI: 39–54), and arterial hypertension (*n* = 98, 60%, 95% CI: 53–68) were the most frequent comorbidities. The most frequent comorbidities by cause of injury were anemia and respiratory diseases (TBI); anemia, arterial hypertension and respiratory infections (stroke); and anemia, respiratory infections and organic heart diseases (post-anoxic encephalopathy). The severity of the comorbidity was rated as mild (86%) in most cases and moderately severe (14%) in the remainder. After evaluating the comorbidity frequency patterns, one category (cerebrovascular diseases) of the original 25 was excluded from further analyses due to low incidence. The frequency of comorbidities finally included in the scale is shown in Figure 3 (frequency of single comorbidities was obtained by combining the scores 1, 2, 3 vs. the score 0).

#### Analysis of Content Validity and Internal Consistency

**Figure 4:**
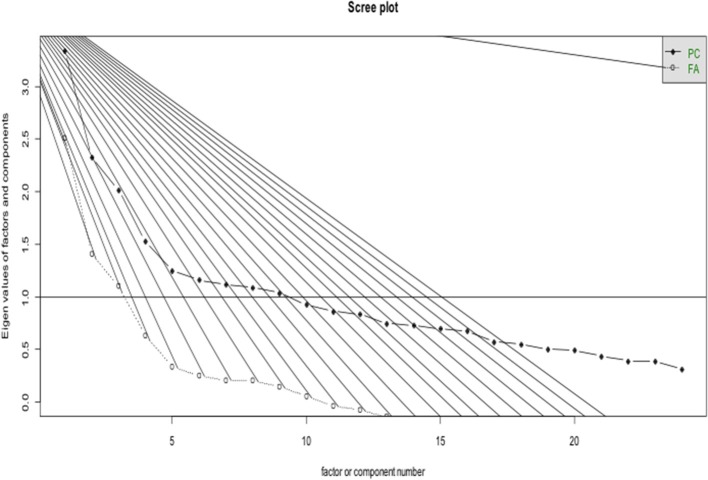
Scree plot for the exploratory factor analysis.

To assess the underlying structure of CoCoS, an exploratory factory analysis (EFA) was performed on the 24-item scale. EFA evaluates the number of distinct constructs needed to account for the pattern of correlations among a set of measures. This is accomplished by explaining a large set of independent variables in terms of a few underlying new variables, called factors. If a variable correlates well with the factor, it is said to load on that factor. By studying the factor loadings, which is interpreted as a correlation coefficient, one can determine how well the factors explain the data. We used the Scree Plot to have an initial estimate of how many factors may underlie the CoCoS (Figure 4). Several exploratory factor models were fit and rotated using a wide array of oblique rotation algorithms. The final EFA solution was based on a varimax rotation and was composed by 12 factors, accounting for 73% of the variance. Table 2 lists all factors and the loading of each variable on each factor.

**Table 2:** Exploratory 12-factor solution of the 24-item scale.

**Item**	**Factor 1**	**Factor 2**	**Factor 3**	**Factor 4**	**Factor 5**	**Factor 6**	**Factor 7**	**Factor 8**	**Factor 9**	**Factor 10**	**Factor 11**	**Factor 12**
Supra-aortic trunks diseases	0.69											
Arterial hypertension	0.64											
Diabetes	0.63											
Anemia		0.76										
Life support devices		0.64										
Pressure ulcers			0.80									
Malnutrition			0.80									
Urinary infections			0.59									
Fractures				0.76								
Hydrocephalus				0.71								
Gastrointestinal diseases					0.74							
Dysautonomia					0.66							
Organic heart diseases					0.42							
Malignancies						0.76						
Renal diseases						−0.52						
Hepatobiliary diseases							0.88					
Seizures								0.86				
Peripheral venous diseases								0.55				
Peripheral artery diseases									0.91			
Rhythm disorders										0.74		
Previous disability										0.70		
Non-infectious respiratory diseases										0.58		
Joint diseases											0.88	
Respiratory infections												0.75

#### Analysis of Reliability and Concurrent Validity

A subsample of the sample investigated above, represented by 52 patients, was assessed for reliability analysis. As reliability analyses, by definition, required repeated assessments in the same patients, a subsample, being representative of the whole sample, was used. Patients were enrolled from two Acute Inpatient Rehabilitation Units (Center 1: *n* = 23; Center 2: *n* = 29). Demographic and clinical characteristics of the sample are reported in Table 3. The most frequent brain injury etiology was stroke (60%), followed by traumatic brain injury (23%), and hypoxic-ischemic encephalopathy (17%). Inter-rater reliability was excellent in both the rehabilitation units [Kw 0.98 (95% CI 0.96–0.99)]. CoCoS total scores correlated significantly with total scores on the Greenfield Scale (ρ = 0.932, 95% CI 0.89–0.96; *P* < 0.0001), demonstrating concurrent validity.

**Table 3:** Demographic and clinical characteristics of patients for the reliability analysis.

**Characteristic**	**Center 1** **(*N* = 23)**	**Center 2** **(*N* = 29)**
Mean age ± SD—year	52.8 ± 20.4	62.34 ± 14.18
Index event—no. (%)		
Stroke	13 (57)	18 (62)
Traumatic brain injury	6 (26)	6 (21)
Anoxic encephalopathy	4 (17)	5 (17)
Clinical diagnosis on admission—no. (%)		
Vegetative state	19 (83)	24 (83)
Minimally conscious state	4 (17)	5 (17)

## Discussion

Comorbidities are common in patients with DOCs and their presence affects prognosis and outcomes (6, 7). Despite these circumstances, a standardized tool for the assessment of comorbidities does not exist. Psychometric analysis of the CoCoS demonstrates that the scale has good content validity and internal consistency, and reliably quantifies comorbidities in patients with coma, VS and MCS. In its present form, the CoCoS can be administered by trained professionals in ~10 min.

**Figure 5:**
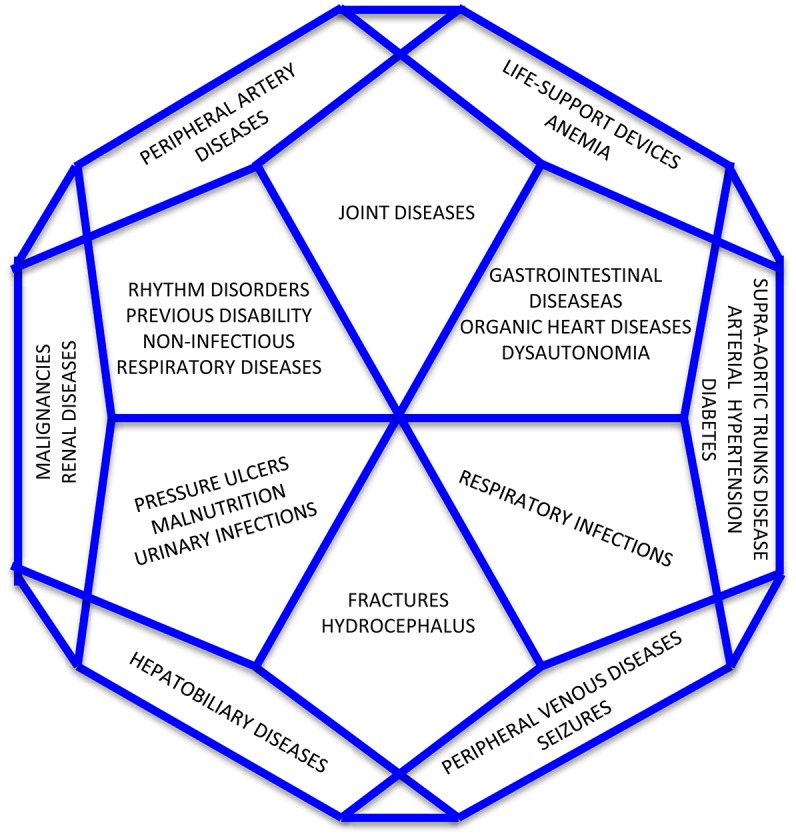
Distribution of comorbidities according the 12-factor solution.

Our findings provide strong evidentiary support for use of the CoCoS in clinical practice and research. Interrater and test-retest reliability measures were excellent, suggesting that the scale is appropriate for identifying comorbidities in daily clinical practice and investigating their influence on prognosis. Factor analysis revealed that a 12-factor solution resulted in the best goodness of fit. This finding suggests that the different comorbidities represented on the scale share a common pathophysiological mileu, and may be explained by the same subfactor (Figure 5). For instance, diabetes, arterial hypertension and supra-aortic trunks disease may represent a sub-factor linked to the vascular profile of patients whereas pressure ulcers, malnutrition, and urinary infections may account for the effects of prolonged immobilization in bedridden patients. Similarly, the presence of fractures and hydrocephalus may identify a sub-factor linked to a specific traumatic profile of patients while the association between life-support-devices and anemia may account for a sub-factor linked to residual instability after severe brain injury. Finally, some comorbidities, such as hepatobiliary diseases and joint diseases, loaded individually on the identified factors, thus appearing as segregated phenomena. In the validation study, we compared the CoCos with the Greenfield Scale, as it is the only available tool previously used in patients with DoC. However, it is noteworthy to mention that the Greenfield Scale is far from the ideal instrument to measure comorbidities in patients with DOCs. It was developed for patients with completely different diagnoses and fails to capture comorbidities specifically associated with DoC. Nevertheless, despite its limitations (e.g., does not include seizures or hydrocephalus), it was the main source of inspiration for the new instrument. Among the older scales, it was the only one that was used in a previous prognostic study in patients with DOC (7). In light of this, the almost perfect agreement found in this study between the two instruments does not diminish the usefulness of the new proposed tool, as the demonstration of equivalence (denoted by the high correlation value) is not a demonstration of insignificance. In fact, although the total scores move in the same direction (as confirmed by the high correlation value between the CoCoS and the Greenfield Total Scores), the informative content of the scale, conveyed by the individual items, is deeply different. The CoCoS is able to provide information about some medical aspects, which are completely neglected by the Greenfield Scale that is more suitable for the assessment of the elderly with age-related diseases. This is in line with the conclusions of our previous published study where we investigated the comorbidities in patients with DOCs through the Greenfield scale: in that occasion, we highlighted the limitations of the use of this tool in the target population as some comorbidities that frequently occur in patients with DOCs were completely missed. Such comorbidities (for instance pressure ulcers, dysautonomia, life support devices, respiratory and urinary infections, hydrocephalus, malnutrition etc…) are likely to be relevant prognostic factors, which are needed to be investigated in prognostic studies. This, in our opinion, justifies the development of a dedicated instrument for the assessment of comorbidities in patients with DOC. The analysis of individual items revealed that almost all patients in the sample required the presence of at least one life-support device. The most frequently-encountered comorbidities were anemia, respiratory infections, urinary tract infections, arterial hypertension, and pressure ulcers. For most items, severity ratings were in the mild to moderate range. These data suggest that even when patients with DOCs are managed in an inpatient rehabilitation setting, many will encounter medical complications requiring use of specific devices (such as the tracheotomy tube, the nasogastric tube or the percutaneous endoscopic gastrostomy, the urinary catheter, the central venous catheter). Evidence from our sample widely reflects this situation, as all but five patients required at least one life-support devices among the aforementioned ones. It was likely that patients without any devices, even those aimed at nutritional support, were fed through parental nutrition at the moment of evaluation due to problems with other nutrition-related devices. Moreover, long-term bedridden patients are likely to suffer from pressure ulcers that, in the most severe form, increase the risk of developing life-threatening sepsis often requiring readmission to the intensive care unit. Similarly, as a result of the prolonged bedridden state, patients may develop severe joint diseases, mainly caused by processes of heterotopic ossification, leading to persistent pain, severe range of motion impairment, and joint deformation. These comorbidities may decrease the probability of functional recovery in patients with DOCs, compromise their access to rehabilitation wards and lead to further deterioration. If not properly identified and managed in the acute or subacute stage of the disease, comorbidities may interfere with long-term functional independence, societal participation and subjective well-being (27). Regarding the prevalence of specific complications, our findings show that anemia is highly prevalent in patients with severe brain injury. This is in line with previous studies suggesting that anemia is frequent after a severe brain injury and that it is associated with poor outcomes even in moderately anemic patients (27–33). Although severity ratings were generally in the mild range and usually did not require blood transfusion, there is evidence that anemia may aggravate the effects of secondary brain injury by reducing cerebral oxygen delivery (28). The optimal level of hemoglobin and the type of transfusion strategy that should be adopted in severely brain injured patients remains unclear (28). Similarly, the role of the arterial hypertension in outcome following severe brain injury remains controversial and there is little agreement on the level of blood pressure thresholds that should be maintained. A recent metanalysis showed that, in patients with post-anoxic encephalopathy, higher blood pressure levels are associated with decreased mortality and better functional outcome (34). On the other hand, the relationship between blood pressure level and functional outcome following TBI is not well-defined. TBI frequently disrupts cerebral autoregulation and may trigger abnormally-high increases in mean arterial blood pressure to maintain optimal cerebral perfusion pressure (35). Moreover, evidence on the development of widespread tissue hypoxia, not confined to regions with the traumatic injury, suggests that excessive lowering of blood pressure levels may not be beneficial in patients with TBI (36, 37). We also found a high prevalence of respiratory and urinary infections which is of some concern as recurrent infections are associated with poor outcome after severe brain injury (7). The frequent occurrence of organic heart diseases and rhythm disorders is consistent with our previous findings suggesting that organic heart disease is a strong predictor of death while rhythm disorders predict failure to fully recover consciousness and demonstrate functional improvement (7). A noteworthy factor, which can influence recovery in disorders of consciousness, is also malnutrition: recent evidence suggests that the type and the timing of nutritional support provided to the patients may affect recovery. Specifically, initiation of nutritional support before 72 h after traumatic brain injury has been reported to be associated with better outcomes in children (38). However, whether the timing and choose of the nutritional route (parenteral or enteral) in adults can reduce the adverse effect of malnutrition on the final recovery of patients is still object of debate and research. Another issue which deserves further investigation deals with the influence of genetic factors on the extent of functional recovery of patients: for instance, growing evidence suggests that specific genetic variations may influence the haematoma enlargement in acute intracerebral hemorrhage, thus affecting the risk of early death and later disability (39–41). Hopefully, in the future also genetic factors will be investigated to obtain even more tailored information on the chances of recovery of patients.

The strengths and level of confidence associated with these findings should be viewed in the context of the study design. We used an iterative process to develop the item content of the CoCoS and relied on investigators with expertise in DOC. Moreover, data collection was conducted in a large sample of patients from different centers. This study has some limitations that should be considered. First, patients with extremely severe comorbidities were poorly represented in our sample, limiting the generalizability of our results to less severe cases. The category of patients in coma is under-represented but our data reflect the situation, which is usually encountered in the Post-Acute Rehabilitative Units, the targeted setting for the use of the proposed assessment tool. In most cases, patients are transferred from hospital intensive care units to post-acute rehabilitative units following the transition from the condition of coma to that of VS or MCS. In a minority of cases patients can be transferred in an early phase, when they are still in a condition of coma. Similarly, patients who emerged from MCS were not represented in the sample due to the relatively early assessment window. Most assessments occurred between 30 and 60 days post-injury, which is typically too early for patients to emerge from MCS. Nevertheless, the scale can be used in the entire spectrum of disorders of consciousness ranging from coma to emergence from MCS. A second limitation is that reliability was investigated in a subsample, which was not fully representative of the whole population. Finally, we didn't check validity by investigating the full scale and the scale subcategories against long-term recovery in level of consciousness and functional outcome of patients. We plan to collect follow-up data on the long-term recovery in level of consciousness and functional outcome of patients in order to test validity in a stronger way and to assess the predictive validity of the CoCoS. These results might allow us to shorten the scale by removing items with poor prognostic power and determine the generalizability of the scale across the continuum of severity.

In conclusion, the CoCoS assess comorbidities in patients with DOCs in a systematic and standardized manner, yielding reliable and valid results. It can be used in different care settings, not only in acute rehabilitation centers but also in intensive care units, neurological or neurosurgical units, and nursing homes. Future studies should explore its prognostic utility in predicting recovery of consciousness and functional recovery, and determine the degree to which outcome is influenced by the primary brain injury vs. subsequent comorbidities.

## Ethics Statement

The research protocol was approved by the Internal Review Board of the University of L'Aquila (no. 16/2019). This was a retrospective study that relied on medical records that included data routinely collected in daily clinical practice. Therefore, we obtained a waiver of consent from the Ethics Committee due to the well-known difficulties in keeping in touch with a large number of patients after considerable time from discharge from the rehabilitative unit. In addition, the data were analyzed and presented in aggregate only. These measures helped ensure that patients' privacy and anonymity were preserved.

## Author Contributions

FP, ACar, SS, YB, MS, and JG contributed to the conception, development, planning of the study and to the manuscript drafting. SG and SK contributed to the revision of the draft. CP, ACas, AD, BC, MS, and GB contributed to the collection of data.

### Conflict of Interest Statement

The authors declare that the research was conducted in the absence of any commercial or financial relationships that could be construed as a potential conflict of interest.
