# Supplementary material for: The Comorbidities Coma Scale (CoCoS): Psychometric Properties and Clinical Usefulness in Patients With Disorders of Consciousness
Source: Front Neurol. 2019 Oct 17;10:1042. doi: 10.3389/fneur.2019.01042 (PMC6812466; doi:10.3389/fneur.2019.01042)
Supplement: Supplementary file 1 [file Data_Sheet_1.pdf]

CoCoS

COMORBIDITIES COMA SCALE

Francesca Pistoia, Antonio Carolei, Yelena G Bodien, Sheldon Greenfield, Sherrie Kaplan, Simona Sacco, Caterina Pistarini, Alfonsina Casalena, Antonio De Tanti, Benedetta Cazzulani, Gianluca Bellaviti, Marco Sarà, Joseph Giacino

Department of Biotechnological and Applied Clinical Sciences, Neurological Institute, University of L'Aquila, L'Aquila, Italy.

1. RESPIRATORY INFECTIONS

0. None
1. Symptoms with or without instrumental and/or laboratoristic signs and/or need for treatment
2. Recurrent infections or treatment-resistant infections
3. Septic shock

☐ Untestable
☐ Previously resolved condition
☐ Newly diagnosed condition
☐ Both previously resolved and newly diagnosed

2. URINARY TRACT INFECTIONS

0. None
1. Positive urine tests (leucocyturia) in the absence of symptoms
2. Positive urine tests (leucocyturia) in the presence of symptoms or clinical signs (fever, haematuria, alterations of the physical characteristics of urine) and need for treatment, or recurrent or treatment-resistant infections
3. Septic shock

☐ Untestable
☐ Previously resolved condition
☐ Newly diagnosed condition
☐ Both previously resolved and newly diagnosed

3. NON-INFECTIOUS RESPIRATORY DISEASE

0. None
1. Currently asymptomatic disease even with a past medical history of obstructive or restrictive diseases
2. Obstructive or restrictive disease, with the need for treatment and well controlled by treatment
3. Obstructive or restrictive disease with recurrent or treatment-resistant episodes of respiratory failure

- [ ] Untestable
- [ ] Previously resolved condition
- [ ] Newly diagnosed condition
- [ ] Both previously resolved and newly diagnosed

4. ORGANIC HEART DISEASE (ischemic or non-ischemic diseases)

0. None
1. Asymptomatic event or previous event without residual symptoms
2. Dyspnea (Respiratory rate>20) and/or suspected angina symptoms, mild or moderate peripheral edema associated with instrumental signs of heart disease (ECG, echocardiogram), condition well controlled by the treatment
3. Cardiogenic shock (systolic BP <90 mmHg, signs of hypoperfusion including oliguria and decreased cardiac output with or without signs of pulmonary edema.

- [ ] Untestable
- [ ] Previously resolved condition
- [ ] Newly diagnosed condition
- [ ] Both previously resolved and newly diagnosed

5. RHYTHM DISORDERS WITHOUT ORGANIC HEART DISEASE

0. None
1. Asymptomatic, only ECG signs
2. Presence of symptoms (e.g. episodes of sustained tachycardia or bradycardia) controlled by the treatment
3. Condition not controlled by the treatment

- [ ] Untestable
- [ ] Previously resolved condition
- [ ] Newly diagnosed condition
- [ ] Both previously resolved and newly diagnosed

6. ARTERIAL HYPERTENSION

0. None
1. Past medical history of arterial hypertension, presence of borderline arterial hypertension without the need for treatment
2. Arterial hypertension well controlled by the treatment
3. Arterial hypertension not controlled by the treatment

- ☐ Untestable
- ☐ Previously resolved condition
- ☐ Newly diagnosed condition
- ☐ Both previously resolved and newly diagnosed

7. DIABETES

0. None
1. Glucose intolerance (100≤glycemia≥125 mg/dL)
2. Diabetes (glycemia≥126 mg/dL) controlled with treatment
3. Poorly controlled diabetes or presence of chronic complications

- ☐ Untestable
- ☐ Previously resolved condition
- ☐ Newly diagnosed condition
- ☐ Both previously resolved and newly diagnosed

8. DYSAUTONOMIA

(Tachycardia>120 beats/min; tachypnea>30 acts/ min, systolic blood pressure> 160 mmHg, hyperthermia or hypothermia, excessive sweating; decerebration or decortication; increased muscle tone; horipilation; flushing)

0. None
1. Positivity of one of the elements of the syndrome
2. Positivity of two or more elements of the syndrome with need for treatment and good control
3. Condition not controlled by the treatment

- [ ] Untestable
- [ ] Previously resolved condition
- [ ] Newly diagnosed condition
- [ ] Both previously resolved and newly diagnosed

9. PERIPHERAL ARTERY DISEASE

0. None
1. Previous peripheral artery disease documented through ultrasound examination or previous bypass surgery
2. Presence of trophic lesions of the skin or instrumental evidence of an abdominal aortic aneurysm
3. Superficial necrosis the need for amputation

- [ ] Untestable
- [ ] Previously resolved condition
- [ ] Newly diagnosed condition
- [ ] Both previously resolved and newly diagnosed

10. SUPRA-AORTIC TRUNKS DISEASE

0. None
1. Stenosis of a single supra-aortic trunk <70%
2. Stenosis of both the supra-aortic trunks <70% or stenosis of a single supra-aortic trunk >70% or history of unilateral carotid endarterectomy
3. Stenosis of both the supra-aortic trunks >70% or history of bilateral endarterectomy

- [ ] Untestable
- [ ] Previously resolved condition
- [ ] Newly diagnosed condition
- [ ] Both previously resolved and newly diagnosed

11. PERIPHERAL VENOUS DISEASE

0. None
1. Uncomplicated varices or a history of thrombophlebitis without sequelae
2. Deep venous insufficiency with mild or moderate peripheral edema or an isolated episode of pulmonary embolism
3. Post-thrombophlebitic syndrome with severe edema or recurrent episodes of pulmonary embolism and/or trophic ulcers of the skin

- ☐ Untestable
- ☐ Previously resolved condition
- ☐ Newly diagnosed condition
- ☐ Both previously resolved and newly diagnosed

12. HEPATOBILIARY DISEASES

0. None
1. Currently asymptomatic disease or previous hepatitis (HBV or HCV), asymptomatic cholelithiasis, previous cholecystectomy
2. Recent hepatitis (<1 year), silent chronic hepatitis
3. Cirrhosis up to the Child B/C stage

- ☐ Untestable
- ☐ Previously resolved condition
- ☐ Newly diagnosed condition
- ☐ Both previously resolved and newly diagnosed

13. GASTROINTESTINAL DISORDERS

0. None
1. Currently asymptomatic disease even with a past medical history of surgery or medical treatment
2. Gastroesophageal reflux disease, symptomatic gastritis, active peptic ulcer, diverticulitis, well controlled by tretament
3. Complications of the above conditions (obstruction, perforation, bleeding)

☐ Untestable
☐ Previously resolved condition
☐ Newly diagnosed condition
☐ Both previously resolved and newly diagnosed

14. RENAL DISEASES

0. None
1. Currently asymptomatic disease or previous kidney disease or interventions for nephrolithiasis (creatinine <1.5 mg/dL)
2. Current uncomplicated kidney disease (creatinine ranging from 1.5-2.5 mg/dL) or nephrolithiasis
3. Complicated kidney disease (creatinine> 2.5 mg/dL), with the need for dialysis or kidney transplantation

☐ Untestable
☐ Previously resolved condition
☐ Newly diagnosed condition
☐ Both previously resolved and newly diagnosed

15. SEIZURES

0. None
1. Sporadic seizures (e.g during fever or metabolic alterations) without the need for antiepileptic treatment
2. Recurrent epileptic seizures with the need for antiepileptic treatment
3. Single or recurrent episodes of status epilepticus

☐ Untestable
☐ Previously resolved condition
☐ Newly diagnosed condition
☐ Both previously resolved and newly diagnosed

16. HYDROCEPHALUS

0. None
1. Normal pressure hydrocephalus
2. Hydrocephalus requiring a ventriculoperitoneal shunt and/or decompressive craniectomy in the acute phase
3. Hydrocephalus requiring urgent decompressive craniotomy after the admission to the acute inpatient brain injury rehabilitation center

- ☐ Untestable
- ☐ Previously resolved condition
- ☐ Newly diagnosed condition
- ☐ Both previously resolved and newly diagnosed

17. FRACTURES

0. None
1. Closed/unexposed and non-displaced fracture
2. Open/exposed or displaced fracture
3. Open/exposed and displaced fracture

- ☐ Untestable
- ☐ Previously resolved condition
- ☐ Newly diagnosed condition
- ☐ Both previously resolved and newly diagnosed

18. PRESENCE OF LIFE-SUPPORT DEVICES (tracheotomy tube, nasogastric tube, percutaneous endoscopic gastrostomy, urinary catheter, central venous catheter)

0. None
1. Only one
2. Combination of two devices
3. > 2 devices

- ☐ Untestable
- ☐ Previously resolved condition
- ☐ Newly diagnosed condition
- ☐ Both previously resolved and newly diagnosed

19. ANEMIA

0. None
1. Mild anemia (Hb 12-10)
2. Moderate to severe anemia (Hb 6-9.9)
3. Severe anemia (Hb<6)

- ☐ Untestable
- ☐ Previously resolved condition
- ☐ Newly diagnosed condition
- ☐ Both previously resolved and newly diagnosed

20. JOINT DISEASES

0. None
1. Asymptomatic disease or presence of sporadic pain; radiographic evidence of degenerative and/or inflammatory disease
2. Persistent pain, mild to moderate range of motion impairment, pain controlled by the treatment, previous replacement of the hip or knee
3. Persistent pain with severe range of motion impairment, severe joint deformation, presence of heterotopic ossification and osteomas.

- ☐ Untestable
- ☐ Previously resolved condition
- ☐ Newly diagnosed condition
- ☐ Both previously resolved and newly diagnosed

21. PRESSURE ULCERS

0. None or intact skin with non-blanchable redness of a localized area usually over a bony prominence
1. Partial thickness loss of dermis presenting as a shallow open ulcer with a red pink wound bed, without slough.
2. Full thickness tissue loss. Subcutaneous fat may be visible but bone, tendon or muscle are not exposed. Slough may be present but does not obscure the depth of tissue loss.
3. Full thickness tissue loss with exposed bone, tendon or muscle. Slough or eschar may be present on some parts of the wound bed.

- ☐ Untestable
- ☐ Previously resolved condition
- ☐ Newly diagnosed condition
- ☐ Both previously resolved and newly diagnosed

22. MALIGNANCIES

0. None
1. Diagnosis and last treatment > 5 years ago
2. Diagnosis and last treatment <5 years ago
3. End stage cancer

- ☐ Untestable
- ☐ Previously resolved condition
- ☐ Newly diagnosed condition
- ☐ Both previously resolved and newly diagnosed

23. MALNUTRITION

0. None
1. Mild condition as inferred by physical parameters alone
2. Moderate condition as inferred by physical parameters and laboratory findings (2.5 g/dl ≤albumin≥ 3.4 g/dl).
3. Severe condition as inferred by physical parameters and laboratory findings (serum albumin < 2.5 g/dl).

☐ Untestable

☐ Previously resolved condition

☐ Newly diagnosed condition

☐ Both previously resolved and newly diagnosed

24. PRESENCE OF PREVIOUS DISABILITY (as a consequence of a previous injury)

0. No previous disability
1. Mild previous disability with reduced autonomy in daily activities without the need for assistance
2. Moderate previous disability with need for assistance in main daily activities
3. Severe previous disability(bedridden patient with bladder and bowel incontinence, totally dependent on others)

☐ Untestable

Untestable item:

In the case the clinical history of the patient it not enough detailed to report information about an item, the examiner should record the score as untestable (UN), and clearly write the explanation for this choice in this box:

………

………

………

………

………

………

………

………

………

………

COMORBIDITIES COMA SCALE (CoCoS)
Record form

Patient:	Diagnosis:	Etiology:

Date of severe acquired brain injury (ABI):	Date of assessment:

**MONTHLY SCORE** (ranging from 0 to 3 as specified in the scale)		Before ABI	Admission	1	2	3	4	5	6	7	8	9	10	11	12
1	RESPIRATORY INFECTIONS														
2	URINARY TRACT INFECTIONS														
3	NON-INFECTIOUS RESPIRATORY DISEASE														
4	ORGANIC HEART DISEASE														
5	RHYTHM DISORDERS WITHOUT ORGANIC HEART DISEASE														
6	ARTERIAL HYPERTENSION														
7	DIABETES														
8	DYSAUTONOMIA														
9	PERIPHERAL ARTERY DISEASE														
10	SUPRA-AORTIC TRUNKS DISEASE														
11	PERIPHERAL VENOUS DISEASE														
12	HEPATOBILIARY DISEASES														
13	GASTROINTESTINAL DISORDERS														
14	RENAL DISEASES														
15	SEIZURES														
16	HYDROCEPHALUS														
17	FRACTURES														
18	PRESENCE OF LIFE-SUPPORT DEVICES														
19	ANEMIA														
20	JOINT DISEASES														
21	PRESSURE ULCERS														
22	MALIGNANCIES														
23	MALNUTRITION														
24	PRESENCE OF PREVIOUS DISABILITY														
**TOTAL SCORE**															

Total cumulative score denotes the cumulative burden of comorbidities according to the following cut-off scores:

0: no comorbidities

1-24: mild comorbidities

25-48: moderate comorbidities

49-72: severe comorbidities

**In the case of missing untestable data, to avoid inaccurate computation of the total cumulative score and inappropriate attribution to one of the three severity categories, total score is not taken into account if more than two variables turn out to be untestable.*
